# Supplementary material for: Recapitulation of Retinal Damage in Zebrafish Larvae Infected with Zika Virus
Source: Cells. 2022 Apr 26;11(9):1457. doi: 10.3390/cells11091457 (PMC9100881; doi:10.3390/cells11091457)
Supplement: Supplementary file 1 [file cells-11-01457-s001.zip › cells-1642998-supplementary.pdf]

Supplementary Table S1. Zebrafish *(Danio rerio)* qPCR primers

Zika virus
1086 FWD: CCG CTG CCC AAC ACA AG
1007 probe: AGCCTACCTTGACAAGCAGTCAGACACTCAA
1162 REV: CCA CTA ACG TTC TTT TGC AGA CAT
Antiviral Immunity:
IFNphi1(a)
FWD: GCTTGGCCGATACAGGATAAT
Probe: TCTGGAGACACCATTTCCAAGCCG
REV: TCCACCTTTGACTTGTCCATC
IFNphi2(c)
FWD: GGCAATTCGTTGCGTTCTTAT
Probe: ACCAACAAACTGCTTCATGCGAAGG
REV: CTCTCAAGTAGACTGTAAGCTGTT
IFNphi3(c)
FWD: GAGGATCAGGTTACTGGTGTTG
Probe: TCAGACACTCCAGAACATTGACGCA
REV: GCCTCTAAAGTCATCCAGTTTCT
IFNphi4(d)
FWD: CTGAGAAGAATGACAGAGCTGAA
Probe: TTTCTGACCATCTCCCAGCTTCTGC
REV: AACATGAGTGTGGAAGGAGAAG
Inflammatory response and granulopoiese:
IL-1
FWD: GCTGGAGATCCAAACGGATAC
Probe: CGGCAGCTCCATAAACACCTTCGA
REV: CTTCATACGCGGTGCTGATAA
TNF
FWD: GGAGGGTGTTTGGGATCATT
Probe: TGCTTCACGCTCCATAAGACCCAG
REV: GTCTCAGCACACTTCCATCTT
IL-6
FWD: AGTTCAGGATGTGGACGTAAAG
Probe: ACCGGCAGAAGTGGCATCTGAT
REV: CTCTCAAACTGCTCGTCTCTG
IL-8
FWD: AGCGCTGTCAGTGCATTA
Probe: AAGTACTCGGACTGAAGGTGACTCCT
REV: GCGATGATCTCCTCGTTTCT
IL-34
FWD: TGCGTTATGAGGAGGTGTTC
Probe: ACTGCGAAACATCAGCAGACTGGT
REV: GACTCACGTAAAGCCACAAATC
Antimicrobial enzymes induced during inflammation:
iNOSa
FWD: CCTAACAATCTCTGTGGTGGAG

Probe: ATGCATTTCGGCACTTGCAGCA
REV: GGGAACTGTGTGTCCTCTTT
iNOSb
FWD: CTGCTCTGTCGACTCTTCATTC
Probe: ACTGAAGAGAAGGCAGAACCTGCA
REV: CCATTTCCAGCTCCACCAATA
NOX1 ($p67^{phox}$)
FWD: GCTCCACTTCAGCCTCAAATA
Probe: AAGTTCCAGAAGTGCTACGGGTGC
REV: AGTCTCTGGAACAAACTCATACAG
NOX2 ($gp91^{phox}$)
FWD: TTAGAGGGTGAAGACGGAGAT
Probe: CCCTGCTCCAAGACTCCAGTGAATT
REV: GTCGTAGAATTGGAGGGAAAGG
Endogenous controls:
EF-1a
FWD: GGCAAGAAGCTTGAAGACAAC
Probe: CCATTGTTGAGATGGTCCCTGGCA
REV: CAAGAGGAGGGTAGGTAGAGAA
GAPDH
FWD: GACGCTGGTGCTGGTATT
Probe: TCTCAACGATCACTTTGTCAAGCTGGT
Rev: GTGCCATCAGGTCACATACA
